# Local knowledge of homegarden plants in Miao ethnic communities in Laershan region, Xiangxi area, China

**DOI:** 10.1186/s13002-024-00676-x

**Published:** 2024-03-18

**Authors:** Jin Luo, Qiang Li, Jianwu He, Jin Yan, Shujie Zhang, Xuepei Chang, Tao Wu

**Affiliations:** 1 College of Biology and Environmental Science, Jishou University, Hunan, 416000 China; 2 National and Local United Engineering Laboratory of Integrative Utilization Technology of Eucommiaulmoides, Jishou University, Hunan, 416000 China; 3 College of Chemistry and Chemical Engineering, Jishou University, Hunan, 416000 China

**Keywords:** Homegarden plants, Local knowledge, Indigenous communities, Functional diversity, Laershan region

## Abstract

**Background:**

Homegardens are small-scale land use systems with significant implications for local livelihoods, biodiversity conservation, and sustainable development due to their diverse flora and fauna. Conducting homegarden surveys enables researchers to gain insights into the traditional ecological knowledge of indigenous communities, preserve homegarden biodiversity, and promote sustainable livelihoods. This study has two objectives: first, to collect, record, and organize data on homegarden plants and related traditional knowledge from three communities in the Laershan Plateau, and second, to analyze the species and functional diversity of homegarden plants in the region while exploring the factors that contribute to the heterogeneous distribution of these plants among the communities of three townships.

**Methods:**

This research employed field surveys in the Laershan Miao Autonomous Region in Xiangxi, China, from September 2021 to August 2023. Data were collected through observation and semi-structured interviews. The study utilized descriptive statistics and quantitative analysis, including the Relative Importance Value (RI), Use Value Index (UV), Jaccard Index (JI), and Comprehensive Index of Land Use Degree (La), to examine the diversity of local homegarden plants and related traditional knowledge, as well as community heterogeneity.

**Results:**

The study area exhibited rich biodiversity, with 152 plant species recorded belonging to 62 families and 124 genera. These findings highlight the importance of homegarden plants in maintaining ecological balance and enhancing system resilience against disturbances. Homegarden plants serve multiple functions such as food, ornamental, medicinal, and fodder purposes, with edible and trade plants having the most abundant knowledge. Furthermore, this research revealed that communities with similar forest resource compositions may have similar homegarden plant compositions, demonstrating the connection between cultural exchange among different communities and patterns of plant species distribution.

**Conclusions:**

The Laershan region boasts diverse homegarden plant species crucial for ecological balance and resilience. Their multifunctionality reflects human impact. Plant diversity varies with residents' lifestyles, needs, and cultural exchanges. The close connection between plants and local life is clear. Factors like resource distribution, cultural exchange, and lifestyle influence plant distribution. Further research is needed for conservation and sustainable development. Future research should focus on culturally valuable plant species and traditional knowledge applications.

## Background

A homegarden is a small land use system that is dominated by humans and includes residences, diverse flora and fauna, and certain structures and landscape features. It provides various services such as food, decoration, medicine, building materials, religion, and ceremonies [1, 2]. Typically, it consists of cottages, trees, and land of varying sizes used for growing flowers, fruits, and vegetables [3]. Known for its rich species diversity [4], its high level of inter and intra species plant genetic diversity makes it a repository of biodiversity [5], which can protect local biodiversity and maintain sustainability [6].

The diverse uses of homegarden plants provide various opportunities for the livelihoods and well-being of local communities. These include self-sustaining food production, commercial cultivation, medicinal plants, fodder, and ornamental purposes. The diversity of homegarden plants is closely related to local livelihoods [7], with most edible plants grown for self-sufficiency [8]. For example, in West Africa, leafy amaranth varieties are harvested and used as seasonal vegetables [9]. Furthermore, many homegardens in different regions also cultivate commercial plants [10]. For instance, coffee is a major commercial plant grown in the homegardens of every household in southwestern Ethiopia, with income from it accounting for 52.43% of the average annual income of wealthy families, 68.27% of middle-income families, and 65.01% of poor families [11]. Moreover, these gardens serve as important repositories of medicinal plants and traditional knowledge [12–14], particularly in remote areas where medicinal plants and traditional knowledge are well-preserved through human management. In Campo Hermoso and Zetaquira, Colombia, bitter plants are referred to as "hot plants," while plants with a sweet flavor are termed "cold plants." The terms "cold" and "hot" express the degree of caution that should be exercised when using these medicinal plants [15]. Homegarden also comprise an essential part of feed and ornamental plants [16, 17]. On the Indonesian island of Sumatra, residents grow large amounts of *Paspalum conjugatum* P.J.Bergius, *Panicum maximum* (Jacq.) B.K.Simon & S.W.L.Jacobs, and *Pennisetum purpureum* (Schumach.) Morrone in their homegardens for their own cattle and sheep feed [18]. Meanwhile, the Salar people in southern China plant various ornamental plants such as *Bougainvillea spectabilis* Willd., *Pelargonium hortorum* L.H.Bailey, *Fuchsia hybrida* Voss, and *Hydrangea macrophylla* (Thunb.) Ser. in their homegardens for decoration and to admire while relaxing [19]. Based on the above reports and for ease of classification, homegarden plants are divided into five types in this study: edible plants, trade plants, medicine plants, forage plants, and ornamental plants.

Homegarden surveys are an effective way to understand indigenous people's traditional knowledge related to biodiversity, as plant diversity and functional diversity are closely related to local people's plant knowledge, which encompasses their understanding of the surrounding environment's plant world [20]. Learning and protecting traditional knowledge related to gardens can promote biodiversity conservation and maintain local livelihoods [19, 21]. Additionally, the reasons for potential differences in homegarden plants and traditional knowledge between different communities need to be further elucidated [22].

The versatile nature of the mentioned homegardens has had a positive impact on the societal economy, ecological environment, and cultural heritage. Evidence indicates that the characteristics and functions of homegarden plants may vary under the influence of different homegarden operators and natural environmental conditions. The diversity in types and structures of homegarden plants is often associated with household traits like land ownership, income level, residential land area, and gardening time, especially notable in tropical and subtropical regions [23]. For example, in certain indigenous communities in Costa Rica, the diversity of edible plants in homegardens is exceptionally rich, encompassing nearly all edible plant resources in the surrounding ecosystem, including rare species [24]. To explore issues concerning the preservation of homegarden plant diversity and associated mechanisms in regions with well-maintained homegardens facing significant environmental changes, it is essential to determine an appropriate landscape scale for investigation. The research focuses on the Laershan region in western Hunan, a typical karst rocky area in southern China heavily influenced by policy-driven land use changes [25]. This area, a traditional settlement of the Miao ethnic group, boasts well-preserved homegardens. Consequently, an extensive field survey of homegarden plants in the Laershan region is conducted to address the following inquiries: What is the current status of traditional ecological knowledge pertaining to homegarden plants that support livelihoods and sustainable development in the Laershan region? What factors influence the distribution of homegarden plants across different townships in the Laershan region?

To address these questions, we conducted field research on homegarden plants and related traditional knowledge in three Miao communities in the Laershan Plateau of western Hunan province, China. The main work of this study included: (1) collecting, recording, and organizing homegarden plants and related traditional knowledge in the local communities; (2) analyzing the species and functional diversity of homegarden plants in this region, and to demonstrate the heterogeneous distribution characteristics in different communities by integrating GIS (Geographic information system) and remote sensing data; and (3) preliminarily elucidating how forest resource distribution and land use changes may affect homegarden plant management and traditional knowledge. This study helps policymakers scientifically understand the dynamics of homegarden plants and related traditional knowledge, allowing them to take appropriate measures tailored to local conditions to promote traditional knowledge protection and homegarden development. This study fills a research gap by investigating homegarden plants in the Miao ethnic region of Xiangxi, China. It presents a systematic catalog of homegarden plants and their associated traditional knowledge. Additionally, it conducts an analysis of the multifunctionality of homegarden plants, considering the local context. The results emphasize the significant role of homegarden plants in fulfilling food needs and supporting livelihoods. Furthermore, it yields preliminary insights into the factors influencing plant diversity in the region, including forest composition, policies, and land use levels. The comprehensive understanding of homegarden plant diversity and cultural significance obtained from this research provides crucial data for protecting and managing local courtyard plant resources. Moreover, it contributes to the preservation and transmission of traditional knowledge and forms a scientific foundation for developing conservation and sustainable utilization strategies.

## Methods

### Study area

**Fig. 1:**
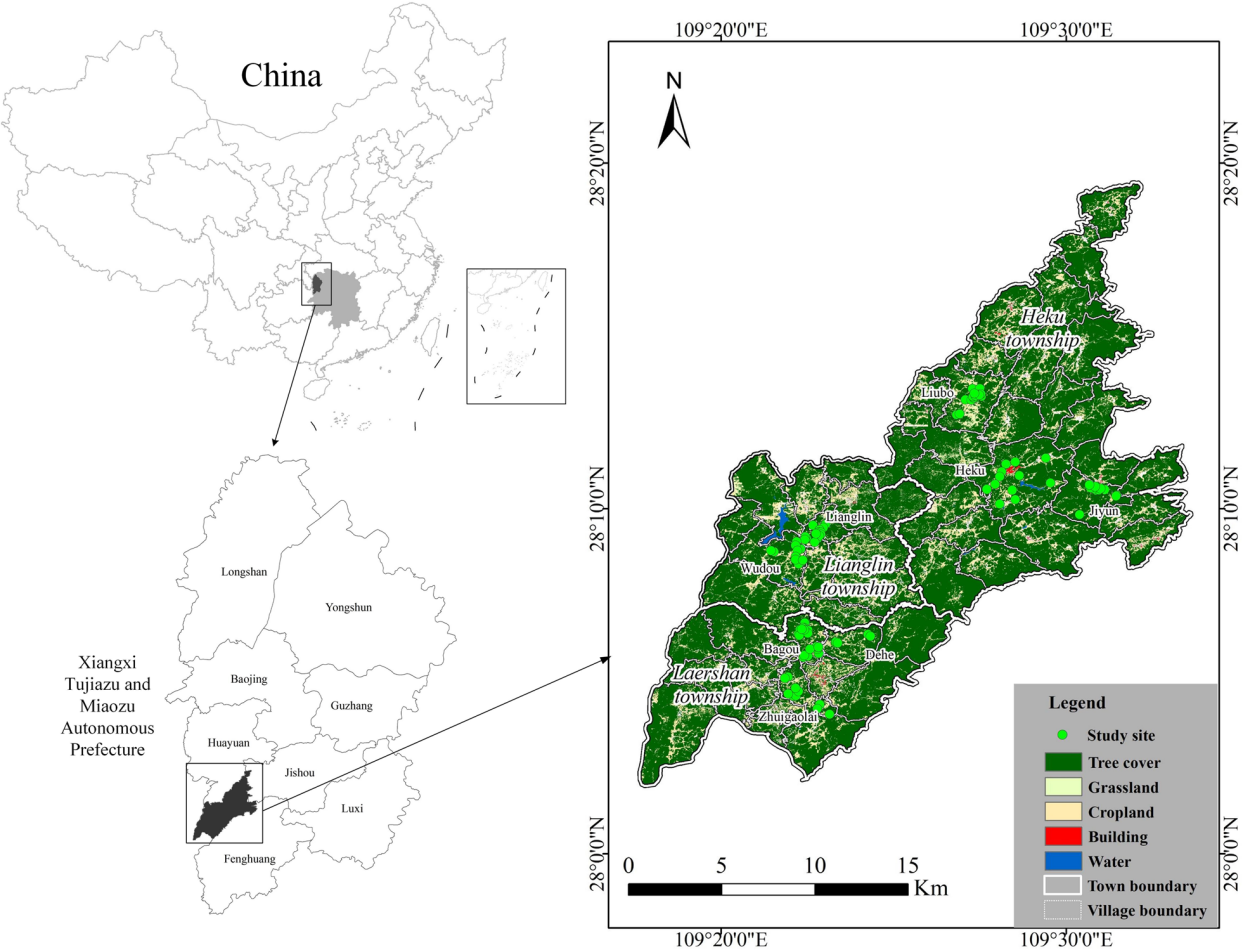
Geographical location of the study area: Laershan Plateau, Xiangxi Tujia and Miao Autonomous Prefecture, Hunan Province, China

The study area is located in the Laershan Plateau in the western part of Hunan Province, China, within the geographical coordinates of 109°18′00″ to 109°33′59″ east longitude and 28°02′00″ to 28°11′31″ north latitude (Fig. 1). The elevation ranges from 700 to 1000 m, with an average annual temperature of 14 °C. The area has a resident population of approximately 53,000 people. The study area is known for its scarcity and infertility of land, complex climate, and diverse small-scale farming practices [26]. Figure 1 illustrates that the scattered red patches labeled as "Building" on the plateau indicate the main settlement areas of the local Miao ethnic group, concentrated in three townships: Laershan, Lianglin, and Heku. These settlements are primarily situated in a handful of villages at elevations ranging from 750 to 860 m, representing some of the lower-altitude zones on the Laershan plateau with advantageous conditions for agriculture and livelihoods.

We chose these study villages because they have a long history of traditional mountain agriculture, and homegarden plants play a vital role in sustaining the daily livelihoods of families. For our field investigation, we selected three Miao ethnic townships in this region, namely Laershan Township, Lianglin Township, and Heku Township. Within these townships, three villages were selected for Heku and Laershan, while Liangliu selected two villages (Table 1).

**Table 1:** Study villages, locations, elevation, and number of households interviewed

Townships	Villages	Geographic location	Elevation (m)	Ethnicity	Language	Total households	Households interviewed	Percent (%)
Laershan	Bagou	109° 23' N, 28° 6' E	830	Miao	Miao	189	14	13.7
	Dehe	109° 23' N, 28° 6' E	812	Miao	Miao	265	10	9.8
	Zhuigaolai	109° 22' N, 28° 4' E	811	Miao	Miao	305	10	9.8
Lianglin	Lianglin	109° 23' N, 28° 9' E	829	Miao	Miao	172	15	14.7
	Wudou	109° 22' N, 28° 8' E	854	Miao	Miao	312	10	9.8
Heku	Liubo	109° 27' N, 28° 13' E	752	Miao	Miao	198	17	16.7
	Heku	109° 29' N, 28° 11' E	795	Miao	Miao	347	12	11.8
	Jiyun	109° 31' N, 28° 10' E	778	Miao	Miao	268	14	13.7
Total						2056	102	100

The vegetation of the Laershan Plateau is primarily composed of tree cover (81.35%), followed by cropland (13.79%). Barren and sparse vegetation accounts for 3.22%, while grassland (1.03%) and water resources (0.32%) are relatively scarce (see Fig. 1) [27]. The main crops in this region include corn, rice, sweet potatoes, and soybeans, which are well-suited to the subtropical monsoon humid climate with hot summers and mild winters. The population is mainly concentrated in township government headquarters and lower-altitude areas, where the local Miao people have long engaged in homegarden farming, resulting in a contiguous homegarden landscape. Knowledge about homegarden plants is still well-preserved to this day.

From a historical perspective, the Laershan region holds a significant position in the continuation of the Miao ethnic group. This area has been the birthplace of almost every Miao rebellion in Chinese history. Since the Ming and Qing dynasties, the central government built the Miao Frontier Wall in this region to isolate the Miao people, especially the term "Sheng Miao" (a term denoting Miao residents in secluded areas like the La'er Mountain plateau, who then faced restricted access to modern resources for production and daily life). This has allowed the Miao people in the Laershan region to maintain their traditional lifestyle.

Up to 2013, the Chinese government implemented a targeted poverty alleviation strategy to lift 70 million Chinese citizens out of poverty [28]. This endeavor entailed investments in sectors like transportation, healthcare, and education across extensive rural areas, including the Laershan region. Consequently, formerly underdeveloped areas started to witness enhanced access to transportation and improved educational prospects. Due to the lack of resources and insufficient food production in historical times, the local small-scale household economy prevailed. In this unique environment, the Miao people developed an adaptable and autonomous homegarden economic system, ensuring their stable livelihood.

**Fig. 2:**
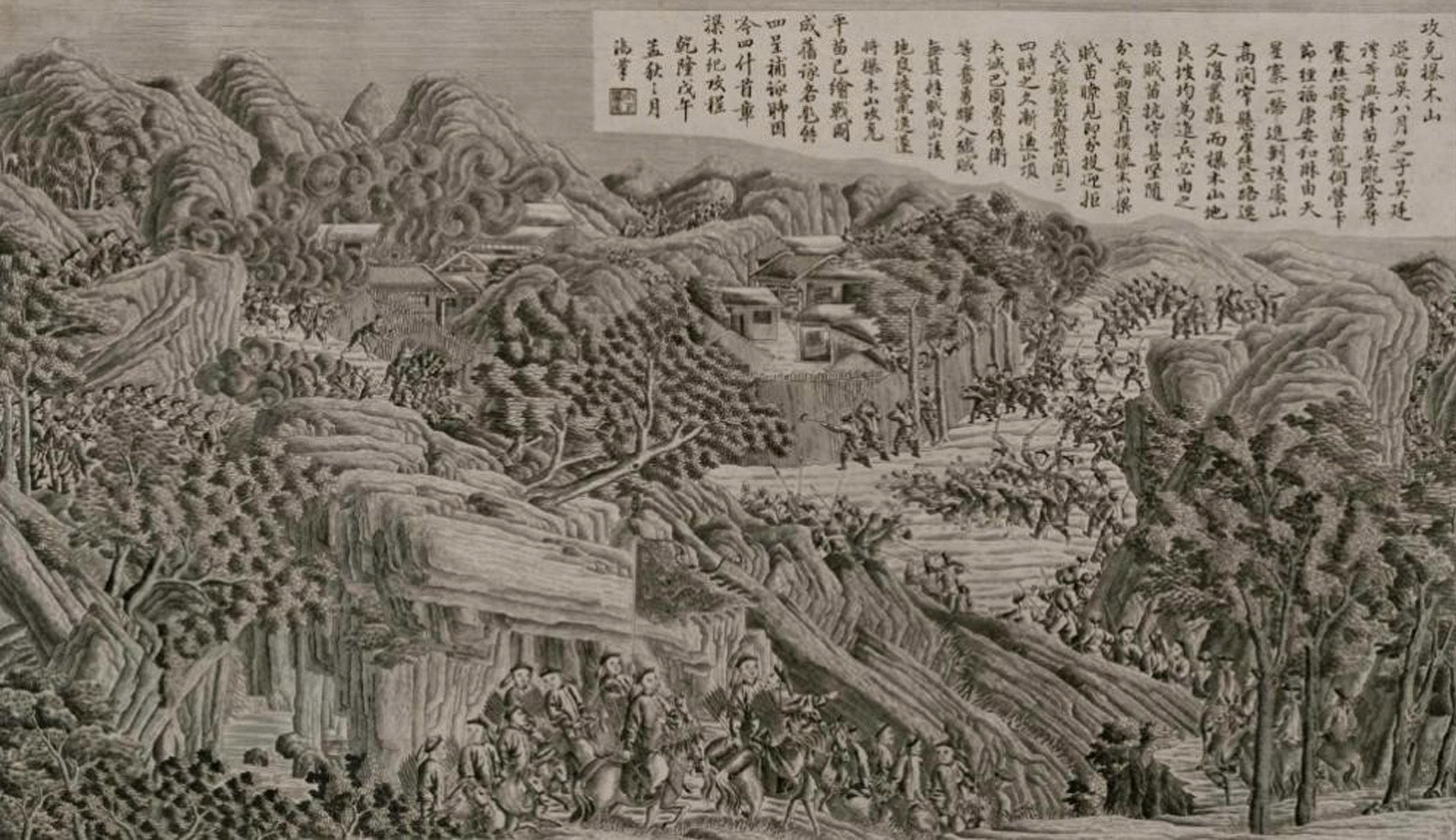
In 1794, the Qing dynasty government dispatched troops to suppress a peasant uprising in the Laer shan region, led by Bayue Wu, also known as Tianban Wu. The accompanying image showcases the homegarden landscape of the Miao people in this area during that period (Courtesy: the Palace Museum of China), depicted through realistic painting techniques by Feng Ning, a court painter of the Qing dynasty

Figure 2 displays a painting depicting the Miao homegarden landscape in the Laershan region in 1794. This artwork (Courtesy: The Palace Museum of China) showcases the living conditions of the Miao people during a politically turbulent period, providing valuable empirical data for studying the homegarden plants in this region.

Overall, due to the influence of factors such as natural geography, vegetation, climate, and historical background, the Laer Mountain region possesses a resilient homegarden plant system with a long history. This system has played a crucial role in sustaining the livelihood and well-being of the local people.

## Data collection

### Ethnobotanical data analysis

The investigation was conducted in the Laershan region from September 2021 to August 2023. A total of eight traditional Miao ethnic villages were randomly selected in the study area, and 102 well-preserved homegardens with traditional management were surveyed on-site (Table 1). The inclusion criteria for selecting gardens in our sample were as follows: (1) proximity to the house; (2) defined boundaries of the land (enclosed by bamboo, bricks, etc.); and (3) exclusion of abandoned land (homegarden of household engaged in continual migrant work without proper maintenance). To ensure sample representativeness and reduce bias, each village included a varying number of homegardens, ranging from 10 to 20.

To optimize the comprehensive documentation of homegarden flora and associated traditional knowledge, this investigation initially employed a semi-structured interview approach to engage with adults overseeing homegarden management on a household level. Given the prevalent limited proficiency in Mandarin among the local Miao population, community administrators and local plant experts, including herbalists and elders knowledgeable in plant utilization, were enlisted to facilitate the translation of Miao language used in daily communication to Mandarin. The participants were then presented with a set of five principal inquiries:

1. Introduction of homegarden plants: Can you provide an overview of the plants cultivated in your homegarden? How many distinct plant species are present?
2. Purpose of cultivation: What is the intended purpose behind cultivating these plants? Specifically, are they cultivated for self-sufficiency (including cooking methods such as steaming, stir-frying, and boiling), trade (mentioned by interviewees as plants intended for sale at markets), medicinal use, ornamental purposes, or as forage for poultry and livestock?
3. Utilization methods: How are these plants utilized, including the processing methods involved?
4. Plant part utilization: Which specific part of the plant is utilized?
5. Origin of plants/seeds: Where were these plants or seeds originally sourced from?

Utilizing semi-structured interviews, we implemented an inventory interview to validate on-site gathered information, encompassing details on plant species, life forms, sizes, uses, and frequency of use. To enhance the precision of the survey data, we eliminated ambiguous species names and rectified inaccuracies. Furthermore, in order to enhance the plant list and associated traditional knowledge, two focus group discussions were organized. These sessions involved botanical experts, local experts in traditional plant usage, and selected information providers. The primary objective was to validate the alignment between local and scientific plant names, establish standardized utilization descriptions, and enhance the compilation of the plant inventory and associated traditional knowledge.

### Ethical considerations

Prior to commencing field surveys and data collection, consent agreements were secured from regional officials and traditional community leaders. Participants provided verbal consent after being briefed on the research objectives. Data collection took place following the receipt of verbal consent. The survey procedures adhered to the ethical guidelines of the International Society of Human Ethology [29].

### Plant identification

During the field investigation, this study captured images of both ubiquitous and locally distinctive plants within the gardens. Additionally, plant specimens that posed identification challenges on-site were collected as voucher specimens. The acquisition of photographic materials and voucher specimens involved securing informed consent and authorization from both the local administrative authorities and garden owners. The identification of plant species primarily relied on the authoritative reference "Flora of China" [30] and relevant botanical websites such as http://www.iplant.cn/. Species name verification was conducted using the database available at https://wfoplantlist.org/plant-list.

### Data analysis

To ascertain the comparative efficacy of plant species in homegardens and the extent of disparities in their utilization across townships, a quantitative assessment was undertaken. This involved the computation of various indicators, namely the Relative Importance Index (RI), Use Value Index (UV), Shannon–Wiener diversity indices, Jaccard Index (JI), and the Comprehensive Index of Land Use Degree. These metrics are detailed below.

### Relative important value (RI)

The Relative Importance Value (RI) is used to evaluate the degree of utilization of homegarden plants in daily life in the Laershan region. This evaluation employs a calculation formula outlined as follows [31]:

$${\text{RI}}={\text{NUC}}+{\text{NT}}$$

where NUC (Number of Use Categories) represents the ratio of a specific plant's use categories to those of the plant with the highest use categories; NT (Number of Types) denotes the ratio of a plant's use categories to the highest recorded in the region. The larger the RI value, the more types of uses and the larger the number of use categories for a certain plant.

### Use value index (UV)

The Use Value Index (UV) [32] assesses the utilitarian value of plants within the local context. The calculation formula for the UV value is expressed as follows:

$${\text{UV}}=\sum \frac{{U}_{{\text{i}}}}{n}$$

In this formula, (*U*i) denotes the frequency with which an individual plant is cited in the utilization information provided by an informant, and (*n*) represents the total number of informants. The UV value spans from zero to infinity, with a higher UV value signifying greater utilitarian value attributed to the plant.

### Jaccard index (JI)

The Jaccard Index (JI) was utilized to assess the similarity of homegarden plants among three townships. By comparing the JI values of homegarden plants in different townships, the extent of similarity in traditional knowledge related to homegarden plant utilization can be evaluated. The formula used to calculate the JI is as follows [33]:

$${\text{JI}}=\frac{c}{\left(a+b-c\right)}*100$$

where "*a*" represents the number of species used by residents in Town A, "*b*" represents the number of species used by residents in Town B, and "*c*" is the number of species used commonly by residents in both Town A and Town B. A higher Jaccard Index (JI) value indicates greater similarity in homegarden plants between Town A and Town B.

### Comprehensive index of land use degree

The Comprehensive Index of Land Use Degree [34] employed to assess the impact of land use changes on traditional knowledge related to homegarden plants in different townships. The formula used to calculate the Comprehensive Index of Land Use Degree is as follows:

$${\text{La}}=100\times \sum_{i=1}^{n}{A}_{i}\times {C}_{i}$$

$${\text{La}}\in \mathrm{100,400}$$

where La represents the Comprehensive Index of Land Use Degree, Ai represents the land use degree classification index of the *i* level, and *C*i represents the percentage of land use degree classification area of the *i* level. The land use degree was classified based on the impact of land use types on homegarden plants, following the method described in literature [34]. The value of La ranges from 100 to 400, reflecting the degree of land use as a continuous indicator. A higher La value indicates a higher local land use degree.

Additionally, the cataloging of homegarden plants and associated traditional knowledge was conducted using Microsoft Office software for statistical analysis. Spatial map visualization and data processing were performed using ArcGIS 10.8 [35]. The geographic data utilized in this study were obtained from various sources including National Geomatics Center of China (https://www.ngcc.cn/ngcc/), Geospatial Data Cloud (https://www.gscloud.cn/), Standard Map Service (http://bzdt.ch.mnr.gov.cn/), and the research conducted by Li et al. [27].

## Results and discussion

### Demography of informants

Informants selected for this study were primarily household heads and the main managers of the homegardens. A total of 112 informants were interviewed, with 84 (75.0%) being male and 25 (25.0%) female. These informants were classified into three age groups, as shown in Table 2. We found that the local residents managing the homegardens tended to be older, with half of the informants being over 50 years old, and farmers accounting for 91.1% of the sample.

**Table 2:** Demographic information about the informants in the study area

Factors	Categories	Number of people	Proportion (%)
Villages	Bagou	15	13.4
	Dehe	11	9.8
	Zhuigaolai	11	9.8
	Lianglin	16	14.3
	Wudou	11	9.8
	Liubo	20	17.9
	Heku	13	11.6
	Jiyun	15	13.4
Age	Less than 30	14	12.5
	30–50	42	37.5
	More than 50	56	50.0
Gender	Male	84	75.0
	Female	28	25.0
Vocation	Farmer	102	91.1
	Government officials	10	8.9

### Species diversity of homegarden plants

The survey recorded a total of 152 plant species, encompassing 62 families and 124 genera. Table 3 provides a summary of the homegarden plant species managed by the respondents, including information on their local common names, scientific names, life forms, uses, and plant parts utilized, use method, UV values, RI values, and cultivation status. The enduring management by local residents has fostered a sustainable and resilient traditional homegarden plant system. This traditional practice has been supported by research on homegarden plant diversity in areas like southwestern China, India, and Thailand [3, 36, 37]. This suggests that the long-term management by local inhabitants has fostered a sustainable and resilient traditional homegarden plant system. Several prevalent plant families discovered in these homegardens include Asteraceae (12 species), Cucurbitaceae (9 species), Fabaceae (9 species), Rosaceae (8 species), and Apiaceae (7 species). For example, plants from the Asteraceae and Cucurbitaceae families exhibit high ornamental value while providing additional advantages such as wind resistance, soil conservation, and climate regulation. Fabaceae plants fulfill the dietary requirements of the local population, and notably, they possess robust nitrogen fixation abilities that enhance soil fertility in the karst region of the Laershan Mountains. Our research aligns with a homegarden plant survey carried out in the Sebeta-Awas District of the Oromia Region in Ethiopia. The prevalent plants in local homegardens belong to the Fabaceae and Asteraceae families. These species play a vital role in providing food to assist local communities in addressing food insecurity. [38]. Despite the locals' potential lack of awareness regarding these beneficial functions during field surveys, their significance should not be overlooked.

**Table 3:** List of homegarden plants in the study area (in alphabetical order)

Voucher codes	Scientific name	UV	RI	Location	Local name	Life form	Family name	Usage	Use part	Use method	Cultivation
LaerHP312	*Acer palmatum* Thunb	0.57	0.65	HK, LES	dou hong feng	Tree	Sapindaceae	Trade, ornamental	Whole plant	Trade it at the bazaar; ornamental	No
LaerHP327	*Achyranthes bidentata* Blume	0.26	0.85	HK	guo rei bei guo zhou	Herb	Amaranthaceae	Edible, medicine, forage	Root, whole plant	Root: crush and apply to bruises to relieve pain; whole plant: feed directly to poultry and livestock	No
LaerHP335	*Acorus calamus* L	0.35	0.65	HK	la zhou cou	Herb	Acoraceae	Medicine, ornamental	Root, stem	Root, stem: the plant is traditional Chinese medicine, when mashed root and stem and applied to the wound for anti-inflammatory and pain-relieving effects; ornamental	Yes
LaerHP371	*Actinidia chinensis* Planch	1.00	0.65	LL	ha bi en	Liana	Actinidiaceae	Edible, trade	Fruit	Fruit: eaten directly after ripening; trade it at the bazaar	Yes
LaerHP355	*Agastache rugosa* Kuntze	0.61	1.10	HK, LL, LES	rei huo xiang	Herb	Lamiaceae	Edible, trade, medicine	Whole plant	Boiling with water to drink can stop vomiting and inflate the stomach and intestines; trade it at the bazaar	No
LaerHP342	*Alangium chinense* (Lour.) Harms	0.39	0.78	HK	dou ming	Tree	Cornaceae	Trade, ornamental	Bark, stem	Bark: weaving ropes; stem: making furniture and ceilings; then trade these at the bazaar; ornamental	No
LaerHP421	*Alcea rosea* L	0.22	0.33	LL	rei ben qin	Herb	Malvaceae	Ornamental	-	Ornamental	No
LaerHP429	*Allium cepa* L	0.91	0.65	LES	bie yang cong	Herb	Amaryllidaceae	Edible, trade	Bulb, leaf	Bulb, leaf: stir-fry and eat; trade bulb and leaf at the bazaar	Yes
LaerHP334	*Allium chinense* G.Don	0.43	0.78	HK, LL, LES	guang jiao dou	Herb	Amaryllidaceae	Edible, trade	Stem tuber	As pickle or stir-fry and eat; trade it at the bazaar	Yes
LaerHP415	*Allium fistulosum* L	1.04	0.98	HK, LL, LES	gong zhong	Herb	Amaryllidaceae	Edible, trade, medicine	Stem, leaf	Stem, leaf: add as a condiment when stir-frying; boiling leaves and drinking water to treat edema; trade stem and leaf at the bazaar	Yes
LaerHP392	*Allium hookeri* Thwaites	0.09	0.33	HK	gong gi cai	Herb	Amaryllidaceae	Edible	Leaf	Stir-fry and eat	No
LaerHP385	*Allium sativum* L	1.26	0.98	HK, LL, LES	gu weng ming en	Herb	Amaryllidaceae	Edible, trade, medicine	Bulb, leaf	Bulb, leaf: stir-fry and eat; the water soaked after crushing can kill insects and sterilize; trade bulb and leaf at the bazaar	Yes
LaerHP326	*Allium tuberosum* Rottler ex Spreng	1.04	0.65	HK, LL, LES	gong gi cai	Herb	Amaryllidaceae	Edible, trade	Tender leaf, stem	Tender leaf, stem: stir-fry and eat; trade tender leaf and stem at the bazaar	Yes
LaerHP404	*Amaranthus tricolor* L	0.70	0.33	LL	rei gan tong	Herb	Amaranthaceae	Edible	Stem, leaf	Stem, leaf: stir-fry and eat	No
LaerHP341	*Anthriscus sylvestris* (L.) Hoffm	0.09	0.33	HK	rei ben	Herb	Apiaceae	Medicine	Root	It’s traditional Chinese medicine, boiling with water to drink can stop coughing	No
LaerHP317	*Apium graveolens* L	0.65	0.33	HK	rei qin cai	Herb	Apiaceae	Edible	Leaf	Stir-fry and eat	Yes
LaerHP333	*Aralia elata* (Miq.) Seem	0.26	0.98	LES	dou duo	Tree	Araliaceae	Trade, medicine, ornamental	Bark	Trade it at the bazaar; bark: its traditional Chinese medicine, boiling with water to drink can cure constipation; ornamental	No
LaerHP365	*Artemisia argyi* H.Lev. & Vaniot	1.22	1.75	HK, LL, LES	hou cai	Herb	Asteraceae	Edible, trade, medicine, forage, ornamental	Tender shoot, whole plant	Tender shoot: stir-fry and eat; whole plant: making moxa sticks for moxibustion, using them as insecticides or fumigation for room disinfection, insecticides,then trade these at the bazaar; whole plant: feed directly to poultry and livestock; ornamental	No
LaerHP350	*Artemisia indica* Willd	0.78	1.30	HK, LL, LES	rei shei guo ao	Herb	Asteraceae	Edible, trade, medicine, forage	Tender shoot, whole plant	Tender shoot: stir-fry and eat; whole plant: making moxa sticks for moxibustion, using them as insecticides or fumigation for room disinfection, insecticides,then feed directly to poultry and livestock	No
LaerHP311	*Artemisia selengensis* Turcz. ex Besser	0.17	1.15	HK, LES	rei shu ao	Herb	Asteraceae	Edible, medicine	Stem, leaf, whole plant	Stem, leaf: stir-fry and eat or as pickled; whole plant: it's a traditional medicine, boiling with water to drink, it has hemostatic, anti-inflammatory, cough relieving, and phlegm resolving effects	No
LaerHP332	*Astragalus sinicus* L	0.83	1.30	HK	rei cao zi	Herb	Fabaceae	Edible, trade, forage, ornamental	Tender leaf, whole plant	Tender leaf: stir-fry and eat; trade tender leaf at the bazaar; whole plant: feed directly to poultry and livestock; ornamental	No
LaerHP370	*Bassia scoparia* (L.) A.J.Scott	0.57	0.98	HK	rei ou zhuo	Herb	Amaranthaceae	Edible, trade, ornamental	Tender stem	Stir-fry and eat; trade it at the bazaar; ornamentalW	No
LaerHP440	*Benincasa hispida* Cogn	1.17	0.98	HK, LL, LES	duo jie	Herb	Cucurbitaceae	Edible, trade, medicine	Fruit, seed	Fruit, seed: stir-fry and eat, and these have anti-inflammatory and swelling reducing effects; trade fruit and seed at the bazaar	Yes
LaerHP460	*Berberis julianae* C.K.Schneid	0.22	0.33	LES	ma xiu dou duo	Shrub	Berberidaceae	Ornamental	-	Ornamental	No
LaerHP450	*Beta vulgaris* L	0.91	0.65	LL, LES	rei dian cai	Herb	Amaranthaceae	Edible, trade	Stem, leaf	Stem, leaf: stir-fry and eat; trade stem and leaf at the bazaar	Yes
LaerHP458	*Biancaea decapetala* (Roth) O.Deg	0.09	0.33	LES	dou duo chui	Liana	Fabaceae	Ornamental	-	Ornamental	No
LaerHP420	*Brassica napus* L	0.52	0.65	HK, LL, LES	rei you cai	Herb	Brassicaceae	Edible, trade	Seed	Extract oil to eat; trade it at the bazaar	Yes
LaerHP414	*Brassica oleracea* L	0.61	0.65	LL, LES	rei bao xin	Herb	Brassicaceae	Edible, trade	Stem, leaf	Stem, leaf: stir-fry and eat; trade stem and leaf at the bazaar	Yes
LaerHP331	*Brassica rapa* L	1.35	0.98	HK, LL, LES	rei bei cai	Herb	Brassicaceae	Edible, trade, forage	Leaf, whole plant	Leaf: stir-fry and eat; whole plant: feed directly to poultry and livestock; trade whole plant at the bazaar	Yes
LaerHP391	*Broussonetia papyrifera (L.) Vent*	0.43	0.98	HK, LL, LES	dou gou	Tree	Moraceae	Edible, trade, ornamental	Staminate flower	Stir-fry and eat; trade it at the bazaar; ornamental	No
LaerHP412	*Buxus sinica* (Rehder & E.H.Wilson) M.Cheng	0.52	0.65	LL, LES	huang yang	Shrub	Buxaceae	Trade, ornamental	Whole plant	Trade it at the bazaar; ornamental	No
LaerHP309	*Camellia japonica* L	0.70	0.90	HK, LES	dou gi	Shrub	Theaceae	Edible, ornamental	Seed, flowers	Flowers: eat directly or stir-fry and eat; seed: pressed for oil; ornamental	No
LaerHP428	*Camellia oleifera* C.Abel	0.83	0.98	LES	dou bi gi	Shrub	Theaceae	Edible, trade, ornamental	Fruit	Pressed for oil; trade it at the bazaar; ornamental	No
LaerHP349	*Capsella bursa-pastoris* Medik	0.39	0.33	HK	rei mia ka	Herb	Brassicaceae	Edible	Whole plant	Stir-fry and eat	No
LaerHP377	*Capsicum baccatum* L	1.09	0.78	HK, LL, LES	ha jiu	Herb	Solanaceae	Edible, trade	Fruit	Stir-fry and eat, add as a condiment when stir-frying; trade it at the bazaar	Yes
LaerHP399	*Castanea mollissima* Blume	0.96	0.65	HK, LL, LES	bi ruo	Tree	Fagaceae	Edible, trade	Fruit	Eating directly; trade it at the bazaar	No
LaerHP457	*Chenopodium album* L	0.17	0.65	HK	li	Herb	Amaranthaceae	Edible, forage	Whole plant, stem, leaf	Whole plant: stir-fry and eat; stem, leaf: feed directly to poultry and livestock	No
LaerHP403	*Chrysanthemum morifolium* Ramat	1.13	1.10	LL	rei ben	Herb	Asteraceae	Trade, medicine, ornamental	Flowers	Trade it at the bazaar; making tea to drink, it can clear heat and detoxifying; ornamental	No
LaerHP330	*Cinnamomum camphora* (L.) J.Presl	0.70	0.98	HK, LL	dou zhang	Tree	Lauraceae	Trade, medicine, ornamental	Branch	Trade it at the bazaar; keep it in the closet, its own odor can repel insects; ornamental	No
LaerHP356	*Cinnamomum cassia* (L.) J.Presl	1.00	1.30	LL, LES	dong gui pi	Tree	Lauraceae	Edible, trade, medicine, ornamental	Bark, stem	Bark, stem: add as a condiment when stir-frying, these have the effect of curing indigestion; trade bark and stem at the bazaar; ornamental	No
LaerHP340	*Cirsium arvense var. integrifolium* Wimm. & Grab	0.09	0.33	HK	rei ci er	Herb	Asteraceae	Edible	Whole plant	Stir-fry and eat	No
LaerHP384	*Citrullus lanatus* (Thunb.) Matsum. & Nakai	1.04	0.65	LES	xi gua	Herb	Cucurbitaceae	Edible, trade	Fruit	Eaten directly after ripening; trade it at the bazaar	Yes
LaerHP456	*Coriandrum sativum* L	0.96	0.78	HK, LL, LES	rei yan xu	Herb	Apiaceae	Edible, trade	Stem, leaf	Stem, leaf: stir-fry and eat, as spices when stir-frying; trade stem and leaf at the bazaar	Yes
LaerHP439	*Cornus controversa* Hemsl	0.30	0.33	LL	dou dai deng	Tree	Cornaceae	Ornamental	-	Ornamental	No
LaerHP449	*Cornus wilsoniana* Wangerin	0.35	0.98	HK	dou rou	Tree	Cornaceae	Edible, trade, ornamental	Fruit	Pressed for oil to eat; trade it at the bazaar; ornamental	No
LaerHP398	*Corydalis racemosa* (Thunb.) Pers	0.09	0.45	HK	rei ben ga chou	Herb	Papaveraceae	Medicine	Whole plant	Apply external application after crushing to treat snake injuries	No
LaerHP338	*Cryptomeria japonica* (Thunb. ex L.f.) D.Don	0.48	0.33	LL	dou gei shei	Tree	Cupressaceae	Ornamental	-	Ornamental	No
LaerHP402	*Cryptotaenia japonica* Hassk	0.91	0.98	HK	rei nuo mou	Herb	Apiaceae	Edible, trade, medicine	Stem, leaf, whole plant	Stem, leaf: stir-fry and eat; whole plant: apply external application after crushing to treat snake injuries; trade whole plant at the bazaar	No
LaerHP366	*Cucumis melo* L	1.17	0.98	HK, LL, LES	duo ding en	Herb	Cucurbitaceae	Edible, trade, forage	Fruit, whole plant	Fruit: eaten directly after ripening; trade fruit at the bazaar; whole plant: feed directly to poultry and livestock	Yes
LaerHP427	*Cucumis sativus* L	1.26	1.18	HK, LL, LES	gua	Herb	Cucurbitaceae	Edible, trade, medicine, forage	Fruit, stem	Fruit: stir-fry and eat; stem: stir-fry and eat, it has anti-inflammatory effects; trade fruit at the bazaar; fruit, stem: feed directly to poultry and livestock	Yes
LaerHP318	*Cucurbita moschata* Duchesne	1.17	1.10	LL, LES	do gun	Herb	Cucurbitaceae	Edible, trade, forage	Fruit, whole plant	Fruit: stir-fry and ea; trade fruit at the bazaar t; whole plant: feed directly to poultry and livestock	Yes
LaerHP419	*Cucurbita pepo* L	1.09	0.98	HK	dou bi hao	Herb	Cucurbitaceae	Edible, trade, ornamental	Fruit	Stir-fry and eat; trade it at the bazaar; ornamental	Yes
LaerHP325	*Daucus carota* L	1.09	0.65	HK, LL, LES	lo bo shei	Herb	Apiaceae	Edible, trade	Root	Stir-fry and eat; trade it at the bazaar	Yes
LaerHP310	*Dioscorea polystachya* Turcz	1.04	0.85	HK, LL, LES	bi shan yo	Liana	Dioscoreaceae	Edible, trade, medicine	Stem tuber, root	Stem tuber, root: stir-fry and eat; trade stem tuber and root at the bazaar	Yes
LaerHP329	*Diospyros cathayensis* Steward	0.70	0.98	HK	duo ga ni	Tree	Ebenaceae	Edible, medicine, ornamental	Fruit	Eaten directly after ripening, able to relieve heart pain; ornamental	No
LaerHP397	*Diospyros japonica* Siebold & Zucc	0.48	0.98	HK	dou man	Tree	Ebenaceae	Edible, trade, ornamental	Fruit	Eaten directly after ripening; trade it at the bazaar; ornamental	No
LaerHP386	*Diospyros kaki* L.f	1.09	0.98	LES	dou man	Tree	Ebenaceae	Edible, trade, ornamental	Fruit	Eaten directly after ripening; trade it at the bazaar; ornamental	No
LaerHP358	*Elaeagnus pungens* Thunb	0.83	1.68	HK, LES	bi gao nia	Shrub	Elaeagnaceae	Edible, trade, medicine, ornamental	Fruit, seed	Fruit: it can be eaten raw, brewed, and boiled with water; seed: eating directly can stop diarrhea; trade fruit and seed at the bazaar; ornamental	No
LaerHP359	*Eriobotrya japonica* (Thunb.) Lindl	1.35	0.98	HK, LL, LES	bi ba	Tree	Rosaceae	Edible, trade, medicine	Fruit, leaf	Fruit: eaten directly after ripening; trade fruit at the bazaar; leaf: eat it will dissolve phlegm and relieve cough	No
LaerHP448	*Eucommia ulmoides* Oliv	0.61	0.90	HK, LL, LES	dou zhong	Tree	Eucommiaceae	Medicine, ornamental	Leaf	Drinking with water can make lower blood pressure, blood lipids, and blood sugar; ornamental	No
LaerHP438	*Fagopyrum acutatum* Mansf. ex K.Hammer	0.65	1.10	HK	rei jiao mai	Herb	Polygonaceae	Edible, trade, medicine	Root tuber	Stir-fry and eat, it can clear heat and detoxify, expelpus and remove blood stasis; trade it at the bazaar	No
LaerHP422	*Ficus carica* L	0.61	0.65	LL	dou ji die bi	Shrub	Moraceae	Edible, ornamental	Fruit	Eaten directly after ripening; ornamental	No
LaerHP316	*Foeniculum vulgare* Mill	0.91	1.10	LES	rei hui xiang	Herb	Apiaceae	Edible, trade, medicine	Tender leaf, fruit	Tender leaf: stir-fry and eat; fruit: add as a condiment when stir-frying; trade fruit at the bazaar	No
LaerHP413	*Galium spurium* L	0.13	0.33	HK	rei sheng xian	Herb	Rubiaceae	Forage	Whole plant	Feed directly to poultry and livestock	No
LaerHP324	*Ginkgo biloba* L	1.22	1.30	LES	dou yin xin	Tree	Ginkgoaceae	Edible, trade, medicine, ornamental	Fruit	Stir-fry and eat as vegetables, it can relieves cough; trade it at the bazaar; ornamental	No
LaerHP372	*Glebionis coronaria* (L.) Cass. ex Spach	0.78	0.65	HK, LL, LES	rei dong hao	Herb	Asteraceae	Edible, trade	Stem, leaf	Stem, leaf: stir-fry and eat; trade stem and leaf at the bazaar	Yes
LaerHP390	*Glycine max* (L.) Merr	1.13	1.23	HK, LL, LES	dei	Herb	Fabaceae	Edible, trade, ornamental	Seed, stem, leaf	Seed: it is used as a cooking raw material, pressed for oil, and used for consumption; trade seed at the bazaar; stem, leaf: feed directly to poultry and livestock; ornamental	Yes
LaerHP383	*Hemerocallis citrina* Baroni	0.57	0.98	LL	rei ben	Herb	Asphodelaceae	Edible, trade, ornamental	Flowers	Stir-fry and eat; trade it at the bazaar; ornamental	No
LaerHP378	*Hibiscus syriacus* L	0.52	0.33	HK, LL	dou ben	Shrub	Malvaceae	Ornamental	-	Ornamental	Yes
LaerHP357	*Houttuynia cordata* Thunb	1.26	1.30	HK, LL, LES	nie rei shu zhou	Herb	Saururaceae	Edible, trade, medicine, forage	Tender rootstem	Stir-fry and eat, it also has the effects of clearing heat and detoxifying; trade it at the bazaar; feed directly to poultry and livestock	No
LaerHP314	*Hydrangea macrophylla* (Thunb.) Ser	0.48	0.33	HK, LL	ben xian hua	Shrub	Hydrangeaceae	Ornamental	-	Ornamental	No
LaerHP315	*Ipomoea aquatica* Forssk	1.04	0.65	LES	rei kong xin cai	Herb	Convolvulaceae	Edible, trade	Stem, leaf	Stem, leaf: stir-fry and eat; trade stem and leaf at the bazaar	Yes
LaerHP367	*Ipomoea batatas* (L.) Lam	1.35	0.98	HK, LL, LES	shuo	Herb	Convolvulaceae	Edible, trade, forage	Root tuber, tender leaf	Root tuber: eating directly; trade root tuber at the bazaar; tender leaf: feed directly to poultry and livestock	Yes
LaerHP328	*Juglans regia* L	1.13	0.65	HK, LL, LES	bi hei dao	Tree	Juglandaceae	Edible, trade	Fruit	Eating directly; trade it at the bazaar	No
LaerHP364	*Kalopanax septemlobus* Koidz	0.26	0.33	LES	dou duo	Tree	Araliaceae	Ornamental	-	Ornamental	No
LaerHP393	*Lablab purpureus* (L.) Sweet	1.00	0.98	HK, LL, LES	ga nong	Liana	Fabaceae	Edible, trade, forage	Fresh pods, whole plant	Fresh pods: stir-fry and eat; trade fresh pods at the bazaar; whole plant: feed directly to poultry and livestock	Yes
LaerHP313	*Lactuca indica* L	0.26	0.65	HK	rei miao you	Herb	Asteraceae	Edible, forage	Stem, leaf, whole plant	Stem, leaf: stir-fry and eat; whole plant: feed directly to poultry and livestock	No
LaerHP396	*Lactuca sativa* L	0.91	0.98	HK, LL, LES	rei wo sun	Herb	Asteraceae	Edible, trade, forage	Stem, leaf	Stem, leaf: stir-fry and eat, feed directly to poultry and livestock; trade stem and leaf at the bazaar	Yes
LaerHP433	*Lactuca serriola* L	0.70	0.65	LL	rei wo sun	Herb	Asteraceae	Edible, trade	Stem, leaf	Stem, leaf: stir-fry and eat; trade stem and leaf at the bazaar	No
LaerHP430	*Lamium amplexicaule* L	0.35	0.45	HK	rei jie song	Herb	Lamiaceae	Medicine	Whole plant	Crushed and apply to the skin to treat external injuries, falls, injuries, redness, and swelling	No
LaerHP441	*Ligustrum lucidum* W.T.Aiton	0.61	0.65	LL	du bei la	Tree	Oleaceae	Medicine, ornamental	Leaf	It is a medicinal herb with antipyretic and analgesic effects; ornamental	No
LaerHP451	*Lilium brownii* F.E.Br. ex Miellez	0.74	1.30	HK	ga chou jio ba lie	Herb	Liliaceae	Edible, trade, medicine, ornamental	Bulb	Stir-fry and eat, it has the effect of clearing heat and reducing swelling; trade it at the bazaar; ornamental	Yes
LaerHP416	*Lilium speciosum* Thunb	0.09	0.65	HK	rei ben qin	Herb	Liliaceae	Edible, ornamental	Bulb	Bulb: stir-fry and eat; ornamental	No
LaerHP423	*Lindera reflexa* Hemsl	0.57	1.55	LES	dou gang	Shrub	Lauraceae	Edible, trade, medicine, forage	Fruit, root, whole plant	Fruit: eaten directly after ripening; root: smashing and applying can stop bleeding, reduce swelling, and relieve pain; whole plant: feed directly to poultry and livestock; trade whole plant at the bazaar	Yes
LaerHP360	*Liquidambar formosana* Hance	0.61	0.65	LL, LES	dou min	Tree	Altingiaceae	Trade, ornamental	Whole plant	Trade it at the bazaar; ornamental	No
LaerHP319	*Lonicera maackii* (Rupr.) Maxim	0.35	0.65	HK	dou bi qin	Shrub	Caprifoliaceae	Medicine, ornamental	Whole plant	It’s traditional Chinese medicine, boiling water and drinking it can dispel rheumatism; ornamental	Yes
LaerHP351	*Loropetalum chinense var. rubrum* Yieh	0.48	0.65	LES	ma xiong dong	Shrub	Hamamelidaceae	Trade, ornamental	Whole plant	Trade it at the bazaar; ornamental	Yes
LaerHP400	*Luffa aegyptiaca* Mill	1.26	1.18	HK, LL, LES	duo nen	Liana	Cucurbitaceae	Edible, trade, medicine, forage	Fruit, leaf	Fruit, leaf: stir-fry and eat, it can clears away heat; trade fruit and leaf at the bazaar; feed directly to poultry and livestock	Yes
LaerHP418	*Malva verticillata* L	0.39	0.33	HK	rei kang nong	Herb	Malvaceae	Edible	Whole plant	Stir-fry and eat	Yes
LaerHP426	*Mentha canadensis* L	0.91	2.00	HK, LL, LES	bo he	Herb	Lamiaceae	Edible, trade, medicine, forage, ornamental	Tender shoot, tender leaf, whole plant	Tender shoot, tender leaf: stir-fry as vegetables, add as a condiment when stir-frying, it can treat colds, fever, sore throat, and headaches; whole plant: feed directly to poultry and livestock; trade whole plant at the bazaar; ornamental	Yes
LaerHP373	*Mirabilis jalapa* L	0.78	1.55	HK, LES	rei ben	Herb	Nyctaginaceae	Edible, trade, medicine, ornamental	Leaf, whole plant	Leaf: stir-fry as vegetables or charge mixture; whole plant: it’s traditional Chinese medicine, boiled with water to drink it can clear heat and dampness, promote blood circulation and regulate menstruation, detoxify and reduce swelling; trade whole plant at the bazaar; ornamental	No
LaerHP405	*Momordica charantia* L	1.30	0.98	HK, LL, LES	dou an	Herb	Cucurbitaceae	Edible, trade, medicine	Fruit	Stir-fry and eat, it has the effect of clearing heat and detoxifying; trade it at the bazaar	Yes
LaerHP434	*Morus alba* L	1.35	1.30	HK	dou liao jin	Tree	Moraceae	Edible, trade, medicine, forage	Fruit, leaf	Fruit: eaten directly after ripening; leaf: boil water and drink to cure cough, feed to *silkworms* or poultry livestock; trade fruit and leaf at the bazaar	No
LaerHP437	*Murraya exotica* L	0.26	0.33	HK, LL	chi li xiang	Tree	Rutaceae	Ornamental	-	Ornamental	Yes
LaerHP368	*Nelumbo nucifera* Gaertn	0.91	1.55	HK, LL	ben huo hua	Herb	Nelumbonaceae	Edible, trade, medicine, ornamental	Stem, seed, leaf	Stem, seed: stir-fry and eat or starch extraction; leaf: soak in water to relieve heat; trade it at the bazaar; ornamental	Yes
LaerHP431	*Nicandra physalodes* (L.) Gaertn	1.22	1.30	HK, LES	an jiao mai	Herb	Solanaceae	Edible, trade, medicine, forage	Seed, root	Seed: add as a condiment when stir-frying, or feed to poultry and livestock; root: regulate the flow of qi to alleviate pain; trade seed and root at the bazaar	No
LaerHP343	*Nicotiana tabacum* L	0.78	0.33	HK	ye	Herb	Solanaceae	Trade	-	Trade it at the bazaar	Yes
LaerHP387	*Oenanthe javanica* DC	1.17	1.43	HK, LES	rei bian gu	Herb	Apiaceae	Edible, trade, medicine, forage	Stem, leaf, whole plant	Stem, leaf: stir-fry and eat; whole plant: it has the functions of clearing heat and dampness, stopping bleeding, and lowering blood pressure; trade whole plant at the bazaar	Yes
LaerHP382	*Opuntia stricta* (Haw.) Haw	0.57	0.33	HK, LL	xian ren zhang	Shrub	Cactaceae	Ornamental	-	Ornamental	No
LaerHP352	*Oreocnide frutescens* (Thunb.) Miq	0.13	0.65	HK	nu mie zuo	Tree	Urticaceae	Trade, medicine	Root, stem, leaf	Root, stem, leaf: trade root, stem and leaf at the bazaar; leaf: it’s traditional Chinese medicine, mashed and apply to wounds can treating injuries	Yes
LaerHP344	*Paeonia* × *suffruticosa* Andrews	0.70	0.33	LES	ben mou dan	Shrub	Paeoniaceae	Ornamental	-	Ornamental	Yes
LaerHP339	*Parthenocissus tricuspidata* Planch	0.48	0.65	LL	la ba yo	Liana	Vitaceae	Forage, ornamental	Stem, leaf	Stem, leaf: feed directly to poultry and livestock; ornamental	No
LaerHP452	*Patrinia monandra* C.B.Clarke	0.17	0.65	HK	rei bai jiang	Herb	Caprifoliaceae	Edible, medicine	Tender leaf	Stir-fry and eat, it has the effect of treating stomach pain	No
LaerHP395	*Perilla frutescens* (L.) Britton	0.96	1.43	HK, LL, LES	rei ca	Herb	Lamiaceae	Edible, trade, medicine, forage	Tender leaf, whole plant	Tender leaf: stir-fry and eat, as spices when stir-frying, it has analgesic and detoxification effects; whole plant: feed directly to poultry and livestock; trade whole plant at the bazaar	No
LaerHP374	*Phaseolus vulgaris* L	0.96	0.65	HK, LL, LES	ga nong	Herb	Fabaceae	Edible, trade	Fresh pods, seed	Fresh pods, seed: stir-fry and eat; trade fresh pods and seed at the bazaar	Yes
LaerHP320	*Photinia* × *fraseri* Dress	0.52	0.65	HK, LES	dou qin miao	Shrub	Rosaceae	Trade, ornamental	Whole plant	Trade it at the bazaar; ornamental	No
LaerHP336	*Photinia serratifolia* (Desf.) Kalkman	0.52	0.33	LL	dou miao qin	Shrub	Rosaceae	Ornamental	-	Ornamental	Yes
LaerHP337	*Phyllostachys heteroclada* Oliv	0.65	1.10	HK, LL, LES	luo ao	Herb	Poaceae	Edible, trade, ornamental	Bamboo shoot, stem	Bamboo shoot: stir-fried food; old stem: weaving bamboo mats; trade bamboo shoot and old stem at the bazaar	No
LaerHP369	*Phyllostachys nidularia* Munro	0.22	0.98	HK, LL	o luo	Herb	Poaceae	Edible, trade, medicine	Bamboo shoot, stem	Bamboo shoot: stir-fried food; old stem: weaving bamboo mats; trade bamboo shoot and old stem at the bazaar	No
LaerHP379	*Phyllostachys reticulata* K.Koch	0.74	1.10	HK	o luo	Herb	Poaceae	Edible, trade, ornamental	Bamboo shoot, stem	Bamboo shoot: stir-fried food; old stem: weaving bamboo mats; trade bamboo shoot and old stem at the bazaar; ornamental	No
LaerHP361	*Phytolacca americana* L	0.17	0.65	HK	jian zhong xiao	Herb	Phytolaccaceae	Edible, medicine	Tender leaf	Stir-fry and eat; whole plant: it's a traditional Chinese medicine	No
LaerHP417	*Pisum sativum var. arvense* (L.) Poir	1.04	0.65	HK	bi wan dou	Herb	Fabaceae	Edible, trade	Seed, fresh pods	Seed, fresh pods: stir-fry and eat; trade seed and fresh pods at the bazaar	Yes
LaerHP436	*Podocarpus macrophyllus* (Thunb.) Sweet	0.48	0.33	LL	dou luo han	Tree	Podocarpaceae	Ornamental	-	Ornamental	No
LaerHP406	*Portulaca grandiflora* Hook	0.39	0.33	HK	da hua ma chi xian	Herb	Portulacaceae	Ornamental	-	Ornamental	Yes
LaerHP345	*Prunus persica* (L.) Batsch	1.13	0.98	HK, LL, LES	bi gua	Tree	Rosaceae	Edible, trade, ornamental	Fruit	Eaten directly after ripening; trade it at the bazaar; ornamental	No
LaerHP401	*Prunus salicina* Lindl	1.13	0.98	HK, LL, LES	bi li	Tree	Rosaceae	Edible, trade, ornamental	Fruit	Eaten directly after ripening; trade it at the bazaar; ornamental	No
LaerHP442	*Pseudognaphalium affine* (D.Don) Anderb	0.09	0.45	HK	rei da guo	Herb	Asteraceae	Medicine	Stem, leaf	Stem, leaf:used as medicine, boiled with water to drink can cough suppressant, expectorant	No
LaerHP447	*Pueraria montana var. lobata* (Willd.) Maesen & S.M.Almeida ex Sanjappa & Predeep	1.13	1.23	HK, LL, LES	li xiu xiu	Liana	Fabaceae	Edible, trade, medicine	Root tuber	Stir-fry and eat; extracted as kudzu powder, boiled with water to drink, it has the functions of relieving external heat, promoting diuresis, quenching thirst, and stopping diarrhea;trade it at the bazaar	Yes
LaerHP446	*Punica granatum* L	1.22	0.98	LES	bi shi liu	Tree	Lythraceae	Edible, trade, ornamental	Fruit	Eaten directly after ripening; trade it at the bazaar; ornamental	No
LaerHP407	*Pyrus pyrifolia* (Burm.f.) Nakai	0.78	0.98	HK, LL, LES	dou bi rua	Tree	Rosaceae	Edible, trade, ornamental	Fruit	Eaten directly after ripening; trade it at the bazaar; ornamental	Yes
LaerHP435	*Raphanus raphanistrum subsp. sativus* (L.) Domin	1.04	1.55	HK, LES	la bo ou	Herb	Brassicaceae	Edible, trade, medicine, forage	Root, seed, whole plant	Root: stir-fry and eat; seed: eating directly can digest and dissolve phlegm; fresh roots: quench thirst and aid digestion; whole plant:feed directy to poultry and livestock; trade whole plant at the bazaar	Yes
LaerHP346	*Reynoutria japonica* Houtt	0.22	0.70	LL	bi dong xiao	Herb	Polygonaceae	Medicine	Stem	Used for medicinal purposes, it has the effects of promoting blood circulation, dispelling blood stasis, unblocking meridians, and relieving cough	No
LaerHP321	*Reynoutria multiflora* (Thunb.) Moldenke	0.65	0.98	HK	bi xi gong	Liana	Polygonaceae	Trade, medicine, ornamental	Root tuber	Trade it at the bazaar; it’s traditional Chinese medicine, boil water to drink can clear away heat and detoxify; ornamental	No
LaerHP424	*Rhododendron simsii* Planch	0.87	0.98	LL, LES	dou ben	Shrub	Ericaceae	Trade, medicine, ornamental	Whole plant	Trade it at the bazaar; it's a traditional Chinese medicine; ornamental	Yes
LaerHP408	*Robinia pseudoacacia* L	0.57	0.53	HK, LL	dou duo	Tree	Fabaceae	Trade, ornamental	Whole plant	Trade it at the bazaar; ornamental	No
LaerHP362	*Rosa chinensis* Jacq	0.78	0.33	HK, LL	xiu duo ben	Shrub	Rosaceae	Ornamental	-	Ornamental	Yes
LaerHP409	*Rubus parvifolius* L	0.83	1.35	HK	bin ge	Shrub	Rosaceae	Edible, trade, medicine	Fruit, whole plant	Fruit: eaten directly after ripening or for brewing and drinking; whole plant: it's a traditional Chinese medicine, boiled with water to drink can analgesic, blood activating, dispelling wind and dampness, and detoxifying effects; trade whole plant at the bazaar	No
LaerHP375	*Rudbeckia hirta* L	0.13	0.33	HK	rei ben	Herb	Asteraceae	Ornamental	-	Ornamental	Yes
LaerHP443	*Rumex japonicus* Houtt	0.13	0.33	HK	bie ga chou	Herb	Polygonaceae	Medicine	Root	It's a traditional Chinese medicine, boiled with water to drink after mashing can treating diarrhea	No
LaerHP388	*Salix babylonica* L	0.61	0.33	HK	dou liao ao	Tree	Salicaceae	Ornamental	-	Ornamental	Yes
LaerHP348	*Sesamum indicum* L	1.13	0.90	HK, LL, LES	ha bi ma	Herb	Pedaliaceae	Edible, trade	Seed	Used as cooking raw materials and dish accessories, pressed oil for serving; trade it at the bazaar	No
LaerHP394	*Solanum lycopersicum* L	1.04	1.10	HK, LL, LES	min la zi	Herb	Solanaceae	Edible, trade, forage	Fruit, leaf	Fruits:stir-fry and eat or eaten as raw fruits; trade fruit at the bazaar; leaf: feed directly to poultry and livestock	Yes
LaerHP322	*Solanum melongena* L	0.96	0.65	LL, LES	guo weng	Herb	Solanaceae	Edible, trade	Fruit	Stir-fry and eat; trade it at the bazaar	Yes
LaerHP432	*Solanum tuberosum* L	0.96	0.65	HK, LES	bi yang yu	Herb	Solanaceae	Edible, trade	Stem tuber	Eating after steaming and cooking; trade it at the bazaar	Yes
LaerHP411	*Spinacia oleracea* L	0.87	0.98	HK	bo cai	Herb	Amaranthaceae	Edible, trade, forage	Leaf, whole plant	Leaf: stir-fry and eat; whole plant: feed directly to poultry and livestock; trade whole plant at the bazaar	Yes
LaerHP425	*Taraxacum mongolicum* Hand.-Mazz	0.87	1.63	HK	ri da tai	Herb	Asteraceae	Edible, trade, medicine, forage, ornamental	Whole plant	Stir-fry and eat, can treat gout; trade it at the bazaar; feed directly to poultry and livestock; ornamental	No
LaerHP453	*Tetradium ruticarpum* (A.Juss.) T.G.Hartley	0.61	0.98	HK	dou za la	Tree	Rutaceae	Trade, medicine, ornamental	Whole plant	The main economic crops in the local area before; it's a traditional Chinese medicine; ornamental	Yes
LaerHP353	*Toona sinensis* (Juss.) M.Roem	0.96	0.65	HK, LL, LES	dou ye	Tree	Meliaceae	Edible, trade	Tender shoot, stem, leaf	Tender shoot, tender leaf: stir-fry and eat; stem: trade tender shoot, stem and leaf at the bazaar	No
LaerHP454	*Trachycarpus fortunei* (Hook.) H.Wendl	0.48	0.98	HK, LL, LES	dou suo	Tree	Arecaceae	Edible, trade, ornamental	Tender flowers	Stir-fry and eat; trade it at the bazaar; ornamental	No
LaerHP363	*Trichosanthes kirilowii* Maxim	0.30	1.48	LL, LES	dou gua lou	Liana	Cucurbitaceae	Edible, trade, medicine	Root, fruit, seed	Root: boiled and eaten, it has the effects of clearing heat, generating fluids, detoxifying and reducing swelling, and is a good contraceptive pill; fruit, seed: it has the effects of clearing heat and phlegm, moistening the lungs and relieving cough, and smoothing the intestines; trade seed at the bazaar	Yes
LaerHP381	*Triticum aestivum* L	0.91	0.65	HK	gou mo	Herb	Poaceae	Edible, trade	Seed	Steaming and eating; trade it at the bazaar	Yes
LaerHP376	*Urtica fissa* E.Pritz. ex Diels	0.43	0.98	HK	rei gei qiang	Herb	Urticaceae	Trade, medicine, forage	Whole plant, stem, leaf	Whole plant: trade whole plant at the bazaar; it's a traditional Chinese medicine, it can treating rheumatic pain; stem, leaf: feed to poultry and livestock	No
LaerHP444	*Vernicia fordii* (Hemsl.) Airy Shaw	0.52	0.98	LL	dou xie dou you	Tree	Euphorbiaceae	Edible, trade, ornamental	Fruit	Pressed for oil; trade it at the bazaar; ornamental	No
LaerHP455	*Vigna unguiculata subsp. sesquipedalis* (L.) Verdc	1.04	0.98	LL	ga nou xing dan	Herb	Fabaceae	Edible, trade, forage	Fresh pods, stem	Fresh pods: stir-fry and ea; trade fresh pods at the bazaar t; stem: feed directly to poultry and livestock	Yes
LaerHP323	*Vitis heyneana subsp. ficifolia* (Bunge) C.L.Li	0.61	0.98	HK	bi gang	Liana	Vitaceae	Edible, trade, ornamental	Fruit	Eaten directly after ripening; trade it at the bazaar; ornamental	Yes
LaerHP380	*Vitis vinifera* L	1.00	0.78	HK, LL, LES	bi gang	Liana	Vitaceae	Edible, trade	Fruit	Eaten directly after ripening or used for brewing wine; trade it at the bazaar	Yes
LaerHP459	*Yucca gloriosa* L	0.35	0.33	LES	feng wei lan	Shrub	Asparagaceae	Ornamental	-	Ornamental	No
LaerHP410	*Zanthoxylum bungeanum* Maxim	1.35	1.30	HK, LL, LES	bi shei	Tree	Rutaceae	Edible, trade, medicine, ornamental	Seed	Add as a condiment when stir-frying, it can ispel wind chill;trade it at the bazaar; it’s a traditional Chinese medicine; ornamental	No
LaerHP347	*Zea mays* L	1.43	1.10	HK, LL, LES	bao mi	Herb	Poaceae	Edible, trade, forage	Seed, whole plant	Seed: steaming and eating or stir-fry and eat; whole plant: feed directly to poultry and livestock; trade whole plant at the bazaar	Yes
LaerHP389	*Zingiber officinale* Roscoe	1.22	1.10	LL	shan	Herb	Zingiberaceae	Edible, trade, medicine	Root, stem	Root, stem: as seasoning, it also has the effects of removing fishy smell, preventing vomiting and nausea; trade root and stem at the bazaar	Yes
LaerHP445	*Zingiber striolatum* Diels	0.35	0.65	LL, LES	bie yang huo	Herb	Zingiberaceae	Edible, trade	Tender shoot, stem tuber	Tender shoot, stem tuber: stir-fry and eat; trade tender shoot and stem tuber at the bazaar	No
LaerHP354	*Ziziphus jujuba* Mill	1.39	1.30	HK, LES	dou bi nei	Tree	Rhamnaceae	Edible, trade, medicine, ornamental	Fruit	Eaten directly after ripening, can replenish energy; trade it at the bazaar; ornamental	No

Based on our survey results, herbaceous plants constituted 56.6% of the total, followed by trees (23.7%), shrubs (12.5%), and vines (7.2%). Herbaceous plants display greater diversity within homegarden ecosystems, potentially attributed to their rapid growth rates, adaptability, and diverse functional attributes that cater to various daily requirements, including medicinal, culinary, and commercial uses. The choice of trees and shrubs may be influenced by economically significant tree species as well as the provision of shade and shelter for housing purposes. Additionally, this composition of life forms among homegarden plants exemplifies the traditional local wisdom pertaining to the sustainable utilization and conservation of plant resources.

### Multifunctionality of homegarden plants

The homegarden plants in the Laershan Plateau exhibit rich diversity while also serving various functions and utilitarian values. This study categorizes the homegarden plants into five primary functional types: edible plants, trade plants, ornamental plants, medicinal plants, and forage plants. The data indicate that there are 106 species of both edible and trade plants, signifying that local homegardens in the area mainly serve the needs of food consumption and livelihood maintenance. By classifying various plant types, we can enhance our comprehension of the importance of local homegardens in fulfilling food needs and supporting livelihoods.

These homegardens also play a significant role in planting ornamental plants for aesthetic enhancement and medicinal plants for health purposes. Certain residents plant various ornamental plants in their homegardens to enhance their happiness. Ornamental plants are easily exchanged between gardens. In the survey, a homeowner in "Dehe Village" discovered beautiful *Yucca gloriosa* in a neighbor's homegarden and transplanted it to their own garden after communication with the neighbor. Medicinal plants play an important role in the Laershan Plateau. For example, *Diospyros cathayensis* is used to treat sudden heart pain, and many indigenous medicinal plants for injuries, snake bites, and other ailments are distributed in homegardens. Homegarden owners who plant these plants usually have traditional knowledge of treating common local diseases. Homegardens have garnered considerable scholarly interest due to their social and cultural significance. Recently, several studies have offered detailed insights into the cultural roles of homegardens, specifically focusing on the traditional practices of local inhabitants in utilizing and preserving natural resources. These studies highlight the transmission and utilization of indigenous traditional knowledge within homegardens [20, 23, 24, 31].

Additionally, we found 30 species of forage plants for animal consumption that locals can collect for home breeding. Through the investigation, we learned that locals have traditionally used plants grown in gardens as animal feed rather than market feed. They have accumulated abundant plant knowledge, such as the fact that long-term feeding of *Galium spurium* to pigs can cause toxicity or even death, but short-term feeding is harmless. Therefore, they alternate feeding *Brassica rapa* and *Galium spurium* to pigs. Accurate identification and effective use of forage plants demonstrate the wisdom and practical knowledge of local inhabitants in harnessing natural resources and managing animal husbandry. Conducting additional research on the attributes and appropriateness of these forage plants is advantageous in mitigating feed shortages and fostering the sustainable advancement of animal husbandry.

**Fig. 3:**
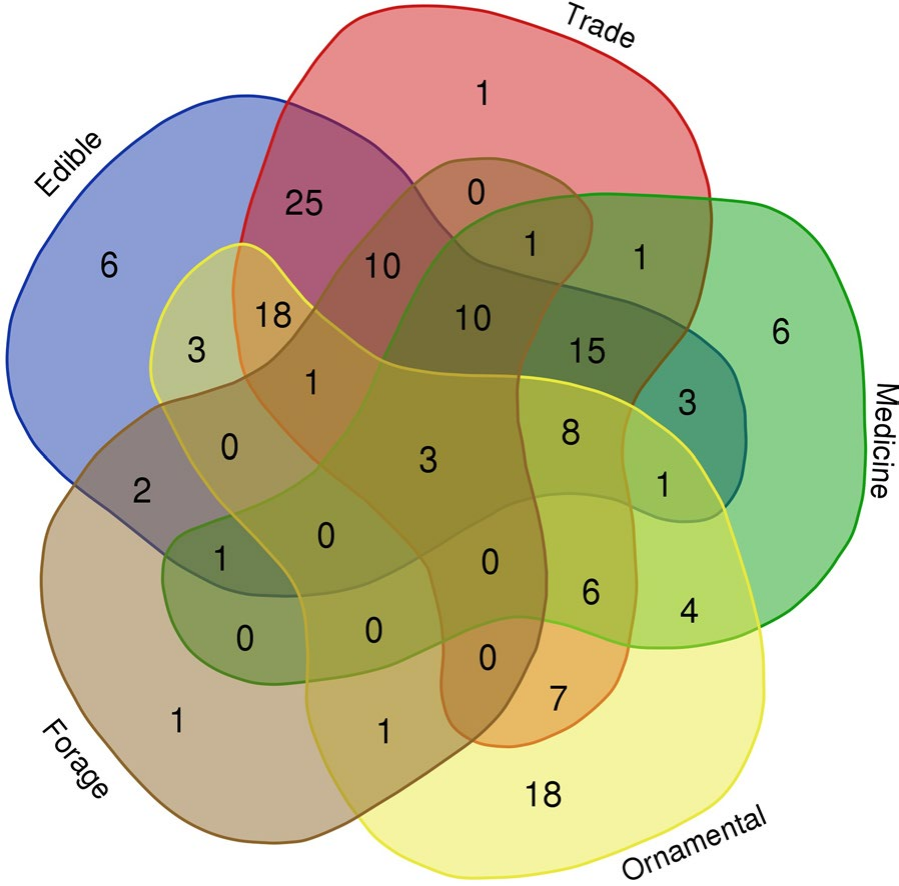
Wayne diagram of plant functional diversity in Miao homegarden on the Laershan Plateau

Figure 3 shows the multifunctionality of some plants among the five categories, and we found that *Artemisia argyi*, *Mentha canadensis*, and *Taraxacum mongolicum* are homegarden plants that exhibit all five functions. Most plants have more than two basic functions. The existence of multifunctional plants reflects the unique traditional knowledge of plant utilization held by local residents. The potential for maintaining resilient homegarden ecosystems, cultural heritage, and high-quality homegarden economic development is evident. Hence, there is a critical need to prioritize the optimization of homegarden planning and management to improve their overall efficiency and sustainability. This is particularly important considering the pivotal role of multifunctional plants in supporting homegarden ecosystems, conserving cultural heritage, and fostering economic development [16, 17, 19].

### RI value

**Fig. 4:**
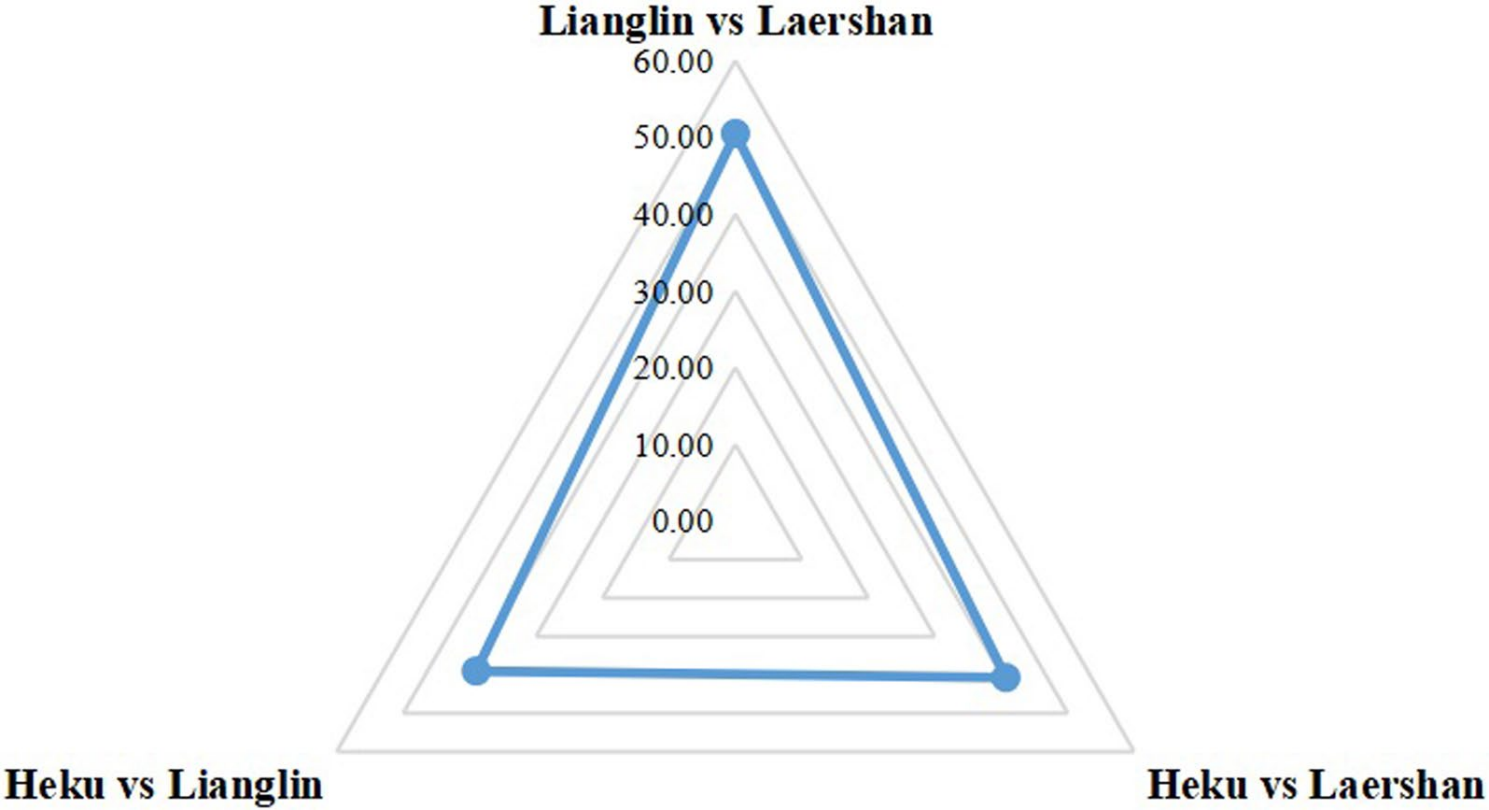
JI value of homegarden plants among three townships

In addition to descriptive statistics on the multifunctionality of homegarden plants, we evaluate the local utilization of each plant based on its RI value. A higher RI value reflects a greater traditional knowledge of the local community regarding a specific plant. Among the surveyed plants, eight species, namely *Mentha canadensis*, *A. argyi*, *Elaeagnus pungens*, *Taraxacum mongolicum*, *Lindera reflexa*, *Mirabilis jalapa*, *Nelumbo nucifera*, and *Raphanus raphanistrum subsp*. Sativus, have an RI value exceeding 1.5. These three plants, *M. canadensis*, *A. argyi*, and *T. mongolicum*, possess all five basic functions (Fig. 4).

The findings highlight the significant traditional knowledge value of these plants in the Laershan region of western Hunan. Our research indicates that these species primarily serve functions related to food provision and sustaining livelihoods. For instance, *M. canadensis* is predominantly employed for consumption and trade, while *A. argyi* is also principally utilized for commercial transactions and monetary exchange. This implies that the prioritization of these plant resources by local residents aligns with the region's production methods and living circumstances.

*M. canadensis* stands out with the highest RI value of 2.00, indicating its wide range of uses and popularity within the local communities. Our investigation uncovered the utilization of its tender shoots and leaves as vegetables or seasonings in a variety of dishes. It is also available for sale in numerous market stalls. Additionally, its beautiful flowers and distinctive aroma are greatly valued. Some residents even brew it as herbal tea to alleviate headaches and cold symptoms. Moreover, it serves as fodder for poultry and livestock. These diverse uses illustrate the deep understanding and extensive knowledge that local residents possess regarding this plant.

*A. argyi*, with an RI value of 1.75, is also widely employed in various domains, potentially attributed to its traditional medicinal applications and the customs of the Miao ethnic group. Our findings indicate extensive trading of this plant in local markets to meet the distinctive cultural needs of the region. The attentive care given to plants with higher RI values in the homegardens indicates a longstanding tradition of utilizing plant resources, fostering the preservation and advancement of traditional knowledge. It also signifies the local residents' reverence and safeguarding of native plant resources.

### UV value

The study aims to analyze the practical value of plants in the local area by ranking them according to their UV value. Table 3 shows that *Zea mays* has the highest UV value (1.43), followed by *Ziziphus jujube* (1.39). Additionally, *Morus alba*, *Zanthoxylum bungeanum*, *Brassica rapa*, *Ipomoea batatas*, and *Eriobotrya japonica* all have a UV value of 1.35, thus ranking them third. Plants with high UV values play a crucial role in the daily life of the local communities. Besides being adaptable and yielding high crops, *Z. mays* also serves as a staple food source for the locals and a primary feed for livestock. *Z. jujube* and *M. alba* fruits are popular and essential components of homegarden planting. Furthermore, *Z. bungeanum* seeds are utilized as a seasoning in local cuisine, and *B. rapa* is a crucial ingredient in pickling. The traditional knowledge of plant utilization by local residents helps recognize their practical value better and provides a foundation for the sustainable use of homegarden plants.

The UV value reflects the practical value of plants and their importance within the local communities. Contrasting plants with higher and lower UV values enables us to identify the preferences and demands of the local communities, obtain insights into the current utilization status of local plant resources, and provide references for the selection and promotion of these plants. For instance, although *B. vulgaris* and *A. hookeri* are non-essential in the local area, they may be more valuable in other regions. In conclusion, these homegarden plants hold substantial ecological, economic, social, and cultural importance. Similar research work, through ranking plants based on their UV values, allows us to observe the geographical specificity of plant resources in different regions, helping us understand the preferences and needs of local communities [13–15, 18]. This approach enables a better comprehension of the role and significance of homegarden plants in local livelihoods.

## Heterogeneous distribution of homegarden plants

The present study conducted a quantitative analysis of the variations in homegarden plant species across three different communities, utilizing the Jaccard Index (JI). A higher JI value signifies a stronger resemblance in homegarden plant species between two villages, whereas a lower JI value implies a greater disparity. The computed results demonstrated that Lianglin Township and Laershan Township achieved the highest JI value of 50.5, which considerably exceeded the values observed between Heku Township and the other two locations. More specifically, the JI value between Heku Township and Laershan Township stood at 40.7, whereas the JI value between Heku Township and Lianglin Township was recorded as 39.0. In summary, the homegarden plant species in Lianglin and Laershan Township exhibit a higher degree of similarity, potentially attributed to frequent exchanges of traditional knowledge between these regions.

The plants with limited utilization and known value, mainly concentrated in the homegarden of Heku Township, include *Malva verticillata* and *Allium hookeri*, which are used for stir-frying vegetables and are only planted by residents of Heku Township. *Podocarpus macrophyllus*, known for its high ornamental value in garden landscapes, is cultivated only by residents of Lianglin Township. *Biancaea decapetala*, adorned with thorns, is planted exclusively by residents of Laershan Township to embellish their homegardens.

All three townships in the study area are inhabited by the Miao ethnic group and situated on the Laershan Plateau. Despite their similar geographical locations and cultural-historical backgrounds, we have noted varying degrees of homegarden plant utilization across these townships. We hypothesize that this variance could be associated with shifts in vegetation types and land utilization practices.

## Factors affecting the heterogeneous distribution of homegarden plants in communities

**Fig. 5:**
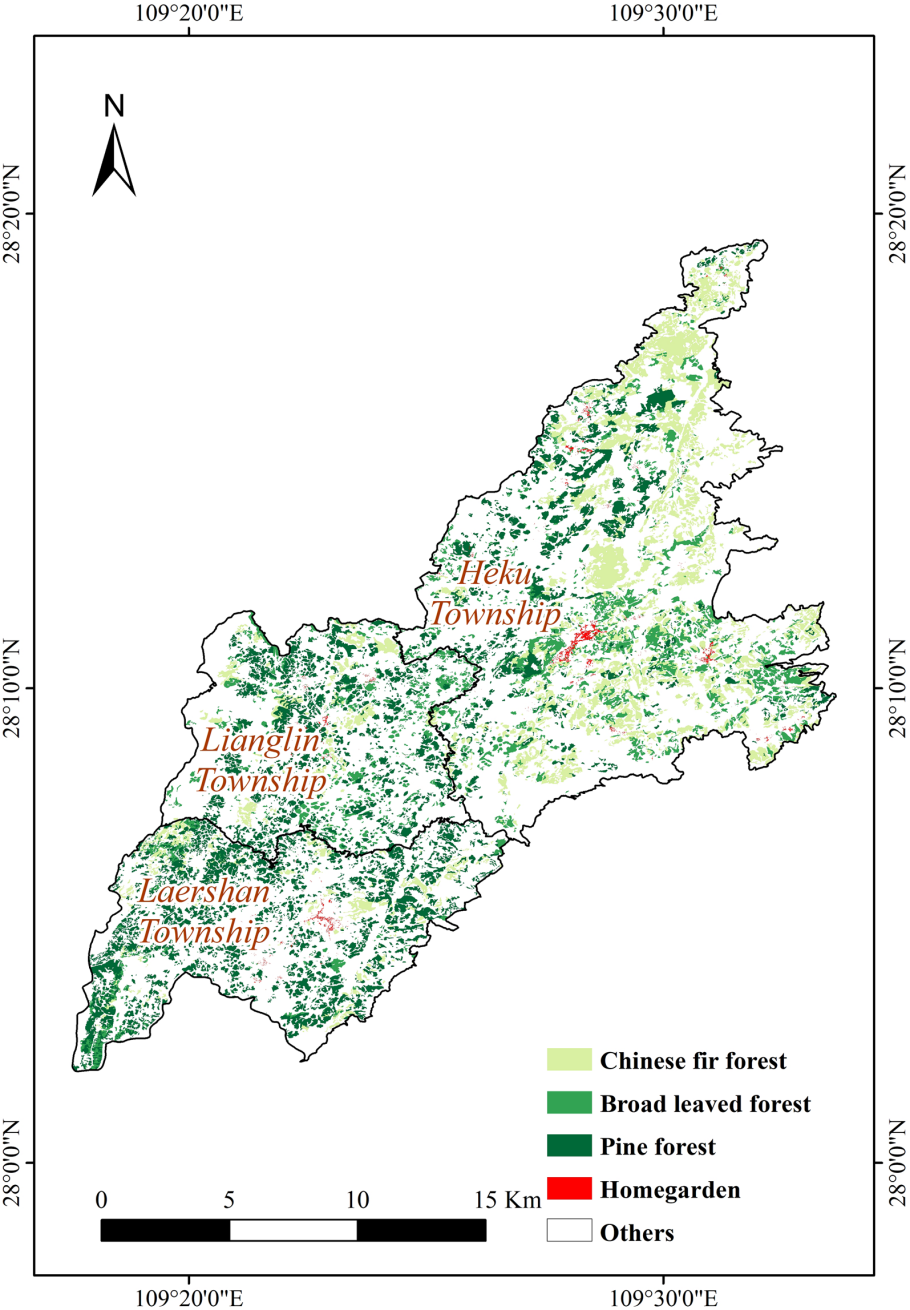
Distribution map of woodland resources in the Laershan Plateau

According to the forest resource distribution shown in Fig. 5, there are significant differences in the composition of forest resources among the three surveyed areas. In Heku Township, Chinese fir forest accounts for the highest proportion, reaching 35%, while broad leaved forest and pine forest make up 13.22% and 12.52%, respectively. In contrast, Lianglin Township has the highest proportion of pine forest at 32.84%, followed by broad leaved forest at 14.12%, and Chinese fir forest with the lowest coverage at 8.72%. In Laershan Township, pine forest covers nearly half of the community area at 48.11%, followed by Chinese fir forest at 7.78%, and broad leaved forest with the lowest coverage at 5.26%. These data indicate substantial differences in the forest resource composition of Heku Township compared to the other two townships. However, the spatial distribution of forest resources in Lianglin and Laershan Township is more similar. This observation aligns with the Jaccard Index, which reflects the diversity of homegarden plants among communities and indicates the significant influence of forest resource distribution on the selection of homegarden plants by local residents. Based on this, we speculate that communities with similar forest resource compositions are likely to have similar compositions of homegarden plants. This finding implies that the forest resource composition may influence the lifestyle and economic development of local residents, impacting the selection and cultivation practices of homegarden plants, along with potential variations in resource utilization patterns among communities. Comparable scenarios have also been identified in Martin's research [39].

**Fig. 6:**
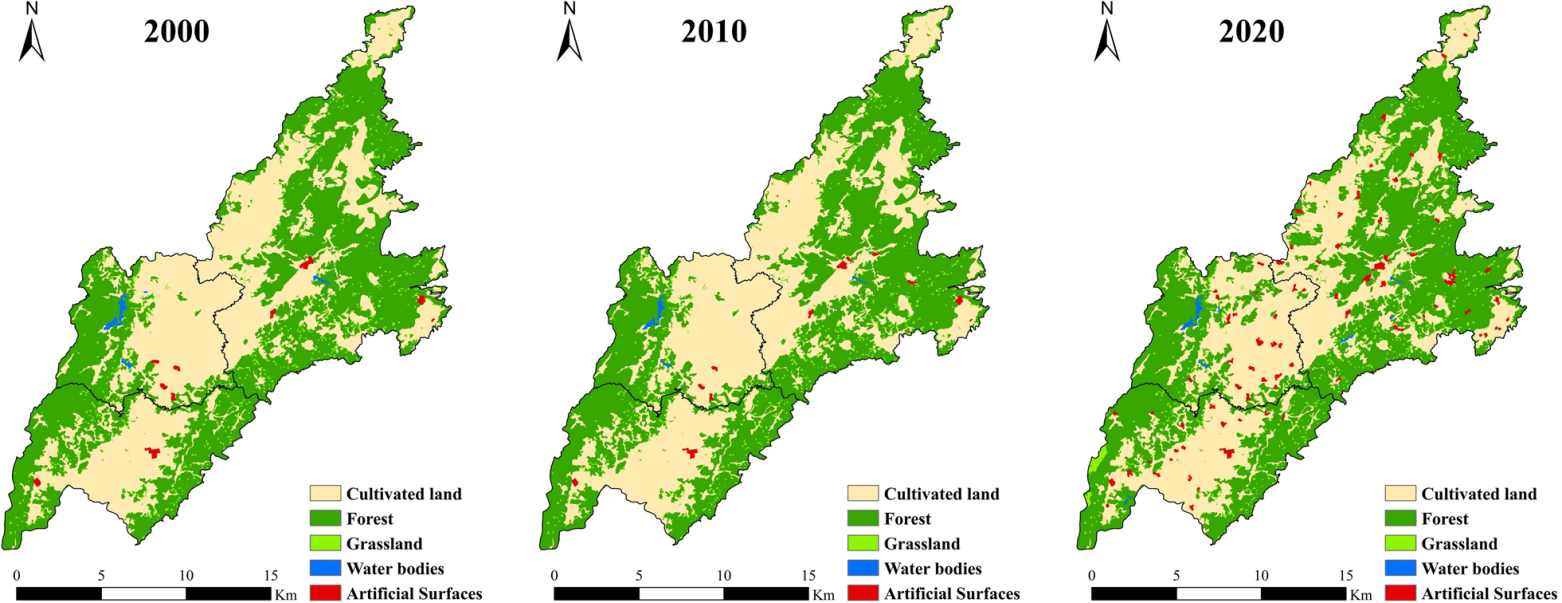
The change map of land use type in the Laershan Plateau

In the three surveyed townships, Heku Township has a significantly higher proportion of buildings (0.41%) compared to Lianglin Township (0.12%) and Laershan Township (0.21%), as observed in the survey data of the three townships. The results of the field surveys reveal that Heku Township has the highest number of homegardens, followed by Laershan Township, while Lianglin Township has the fewest. Due to the prevalence and good maintenance of buildings in the study area, they serve as an effective representation of homegarden spatial distribution, which is consistent with the findings of the field surveys (Fig. 5). Furthermore, the number of homegardens not only reflects the degree of focus on plant diversity and conservation awareness but also signifies the transmission of traditional knowledge [40]. Moreover, simulation results provide further evidence of the strong correlation between homegardens and traditional knowledge [41]. Consequently, it is hypothesized that the quantity and area of homegardens in an area are directly proportional to the extent of traditional knowledge it possesses (Fig. 6).

During the interviews conducted in Laershan Township, it was found that limited transportation hindered interactions between communities in the past, resulting in a restricted dissemination of knowledge about homegardens and wild plants. Most residents focused on the economic benefits brought by farming and neglected the management of homegarden plants. However, improved roads have strengthened communication between communities, leading to the widespread dissemination of knowledge about homegardens and wild plants. This has promoted plant diversity and renewed recognition of the economic value of homegardens among residents. To analyze this change in depth, we collected land use data for Laershan Plateau in 2000, 2010, and 2020 and analyzed the changes in land types related to homegarden management. A comparison revealed no significant changes in land use types from 2000 to 2010. However, by 2020, there was an increase in artificial surfaces, a decrease in cultivated land, minimal changes in water bodies, and an increase in forest and grassland areas. These changes align with the "returning farmland to forest" policy implemented by the local government between 2010 and 2020 [28], as mentioned in the interviews. Therefore, we hypothesize that transportation accessibility and policy orientation are key factors influencing homegarden plant diversity.

According to our analysis of the comprehensive index of land utilization intensity (La) in Table 4, there was almost no change in the land utilization intensity of the entire survey area or the three individual communities between 2000 and 2010. However, compared to the past, there was an improvement in land utilization intensity in 2020, primarily reflected in a significant increase in forest and artificial surfaces. The expansion of forest areas provided more space for wild edible plants (WEPs), which are rich in nutritional and medicinal value and hold significant importance for the lives and health of residents [42, 43].During field investigations, residents expressed that the implementation of the rural revitalization strategy has provided them with more time and energy to learn about plant knowledge and take care of their own homegardens, as their material lives have been guaranteed. Therefore, plants with various uses and knowledge acquired through knowledge exchange were discovered in the wild and planted in their own homegardens. The increase in artificial surfaces means that more homegardens are being maintained, thereby enhancing the diversity of homegarden plants [44].

**Table 4:** Comprehensive index of land use degree (La) of Laershan

Year	Heku township	Lianglin township	Laershan township	All study area
2000	259.04	240.61	257.85	254.54
2010	259.10	240.08	257.64	254.40
2020	263.95	247.95	259.65	259.22

Our analysis reveals a positive correlation between the richness of homegarden plants and related traditional knowledge with land utilization intensity. The highest level of land utilization intensity in 2020 was observed in Heku Township, which also had the highest homegarden plant diversity. Conversely, Lianglin Township, with the lowest land utilization intensity, had the lowest homegarden plant diversity. In other words, higher land utilization intensity is associated with greater richness of plants and related traditional knowledge, while lower land utilization intensity is linked to lower richness of plants and related traditional knowledge. This finding contributes to a better understanding of the ecological and cultural diversity in rural areas, as well as the impact of policy promotion and improved transportation conditions on homegarden plant diversity. This study revealed that shifts in land use have impacted the utilization of homegarden plants, affirming the substantial role of enhanced land policies and infrastructure in promoting local ecology and culture [45, 46]. Further attention and research on this phenomenon can provide strong support for the rural revitalization strategy and the construction of an ecological civilization, promoting sustainable development in rural areas.

## Conclusion

This study recorded 152 plant species from 62 families and 124 genera, which play a crucial role in maintaining ecological balance and enhancing the resilience of homegarden ecosystems against disturbance in Miao ethnic communities in Laershan Region. Their multifunctionality, including use for food, trade, ornamentation, medicine, and fodder, highlights the significant impact of human activities on the diversity of homegarden plant species. The diversity of homegarden plant species varies among the three communities in the Laershan region, which may be influenced by the selection of plants from similar forest resource communities.

This reflects the different lifestyles and needs of residents as well as the correlation between cultural exchange among communities and distribution patterns of plant species. The residents of the Laershan Plateau use plants based on their lifestyles and needs, demonstrating the close connection between homegarden plant species and the local residents' way of life. Multiple factors influence the heterogeneous distribution of homegarden plants, including resource distribution, cultural exchange, natural conditions, and lifestyle. Future research should further investigate these factors to provide a theoretical basis for the conservation, utilization, and sustainable development of homegarden plant resources.

Although this study provides a systematic understanding of the diversity of homegarden plant species in the Laershan Plateau, the time span was relatively short, limited to the years 2000, 2010, and 2020, and may not fully demonstrate long-term trends in analyzing the relationship between changes in land use types and plant diversity. Future research should consider extending the time frame to obtain a more comprehensive understanding.

This paper offers a preliminary exploration of the connection between plant species diversity and traditional culture. Future research should further investigate this topic to reveal more culturally valuable plant species and the applications of these plants in traditional knowledge and practices.

## Data Availability

The data for this study may be availed upon request.

## Abbreviations

GIS: Geographic information system
RI: Relative importance index
UV: Utilization value
JI: Jaccard index
La: Comprehensive index of land utilization intensity
WEPs: Wild edible plants
